# Evaluation of Stiffness Changes in a High-Rise Building by Measurements of Lateral Displacements Using GPS Technology

**DOI:** 10.3390/s131115489

**Published:** 2013-11-13

**Authors:** Se Woon Choi, Ill Soo Kim, Jae Hwan Park, Yousok Kim, Hong Gyoo Sohn, Hyo Seon Park

**Affiliations:** 1 Department of Architectural Engineering, Yonsei University, Seoul 120-749, Korea; E-Mails: watercloud@yonsei.ac.kr (S.W.C.); yskim1220@yonsei.ac.kr (Y.K.); 2 POSCO A&C. Co., Ltd., Seoul 135-080, Korea; E-Mail: grandbluesea@korea.com; 3 Dongbu Corporation, Seoul 135-523, Korea; E-Mail: jaehwan@dongbu.co.kr; 4 Department of Civil Engineering, Yonsei University, Seoul 120-749, Korea; E-Mail: sohn1@yonsei.ac.kr

**Keywords:** GPS, high-rise buildings, stiffness, lateral displacement, outrigger

## Abstract

The outrigger truss system is one of the most frequently used lateral load resisting structural systems. However, little research has been reported on the effect of installation of outrigger trusses on improvement of lateral stiffness of a high-rise building through full-scale measurements. In this paper, stiffness changes of a high-rise building due to installation of outrigger trusses have been evaluated by measuring lateral displacements using a global positioning system (GPS). To confirm the error range of the GPS measurement system used in the full-scale measurement tests, the GPS displacement monitoring system is investigated through a free vibration test of the experimental model. Then, for the evaluation of lateral stiffness of a high-rise building under construction, the GPS displacement monitoring system is applied to measurements of lateral displacements of a 66-story high-rise building before and after installation of outrigger truss. The stiffness improvement of the building before and after the installation is confirmed through the changes of the natural frequencies and the ratios of the base shear forces to the roof displacements.

## 1. Introduction

Construction of high-rise buildings for both business and residential purposes has increased in pace with the progress of construction technology. Structural design for high-rise buildings consists of strength design for assuring the safety of the overall structure or its members, and stiffness design to satisfy the limits of the maximum lateral displacement at the top of the building and inter-story drifts. Owing to the tallness and high slenderness ratio of a high-rise building, the relative importance of stiffness design is increasing [1–7].

Although the limit on the maximum lateral displacement of a high-rise building under wind load, which is one of the representative indices of stiffness design, is not provided as a standard, it is usually designed and implemented in construction to be in the range 1/400–1/600 of the building height in general. Actual maximum displacement of an existing high-rise building is a direct assessment index on its stiffness and can be further utilized as an important data for assessing the building's safety and serviceability.

Measurements of structural responses of high-rise buildings based on accelerometers, which are small and light, allow relatively accurate evaluation of horizontal accelerations under lateral loads. However, it has such difficulties as problems in displacement reference point setting, accumulated errors from double integration, noise introduction from extended cable connection to storage device, and maintenance problems of cable and measurement devices [8,9]. Therefore, this method is considered rather difficult to apply for measuring relative displacements of high-rise buildings consisting of static and dynamic fluctuating displacements.

As an alternative to the method based on accelerometers, numerous studies have been conducted to demonstrate the feasibility of GPS for measurements in various fields [10–14]. In the case of buildings and infrastructures, there have been diverse studies on measuring of the maximum lateral displacements and monitoring of movements of slender building structures, including high-rise buildings [15–20].

In the structural design of high-rise building, the maximum lateral displacement of a high-rise building must be checked not to exceed a specific limit since an excessive lateral displacement can cause the failure of structural or non-structural elements. For this reason, the amount of material required for a high-rise building is determined by the selection of lateral load resisting system. The efficiency of structural design for a high-rise building is tested by the amount of material used in the system. The outrigger truss system is one of the most frequently used lateral load resisting structural systems. In the outrigger system, external columns are tied to a central core wall with very stiff outriggers [7]. If a high-rise building is subjected to lateral loads, the stiff outriggers supported by external columns resist rotation of the central core. Then, the lateral stiffness of a high-rise building is improved by increasing the effective width of a high-rise building. However, little research has been reported on the effect of installation of outrigger trusses on improvement of lateral stiffness of a high-rise building through full-scale measurements.

In this paper, stiffness changes of a high-rise building due to installation of outrigger trusses have been evaluated by measuring lateral displacements using GPS. To confirm the error range of the GPS measurement system used in the full-scale measurement test, the GPS displacement monitoring system is investigated through a free vibration test of the experimental model. Then, for the evaluation of lateral stiffness of a high-rise building under construction, the GPS displacement monitoring system is applied to measurements of lateral displacements of a 66-story high-rise building before and after installation of outrigger trusses.

## 2. Preliminary Measurement Tests Using the Experimental Model

The carrier-phased based differential global positioning system (CDGPS) allows improved point positioning by establishing a reference station at a known position, comparing the accurate position of the reference station and the observed position value by GPS receiver to identify errors, and then broadcasting the error information to measuring stations in the area. CDGPS allows accurate positioning by considering such common error components as satellite orbit error, satellite clock error, ionosphere and troposphere time delay, and eliminating them.

A lot of studies on the accuracy tests of GPS have been proposed. The accuracy of GPS can be assessed by using a motion simulator [17,21–25] or by comparing GPS and accelerometer data [26,27]. The measurement accuracy of GPS has also been evaluated by using the data measured during non-motion as well as the data measured during motion. It is demonstrated that GPS can measure the displacement accurately within a certain amplitude and frequency range [21,23].

**Figure 1.**
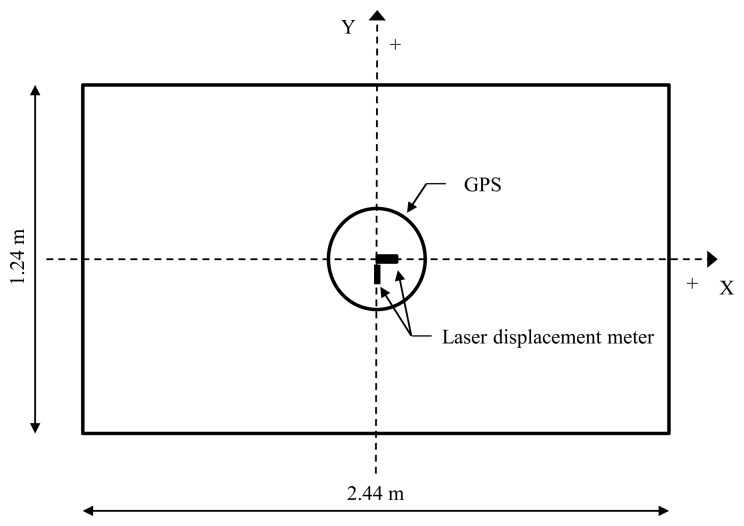
Accuracy test scheme for the CDGPS.

To confirm the error range of the GPS measurement system used in this study (*i.e.*, full-scale measurement test), we investigated the feasibility of the displacement measurement using CDGPS by artificially generating displacements on the test model and comparing the GPS-measured displacements against actual laser measurements. As shown in Figure 1, we installed a GPS receiver and a laser displacement meter to compare displacement histories by two measurement devices in free vibration. According to the specifications of the Trimble 4,700 receiver [28] (Trimble, Sunnyvale, CA, USA), which was used as a GPS receiver in this study, the standard deviations of the data measured with this system for baseline of 10 km or less show horizontal error of 1 cm + 2 parts per million (ppm) and vertical error of 2 cm + 1 ppm. A Keyence LB-301 unit (Keyence, Osaka, Japan) with a measurement range of ±10 cm, maximum sampling rate of 915 Hz, and a linearity of 0.32 mm was employed as a laser displacement meter [29].

**Figure 2.**
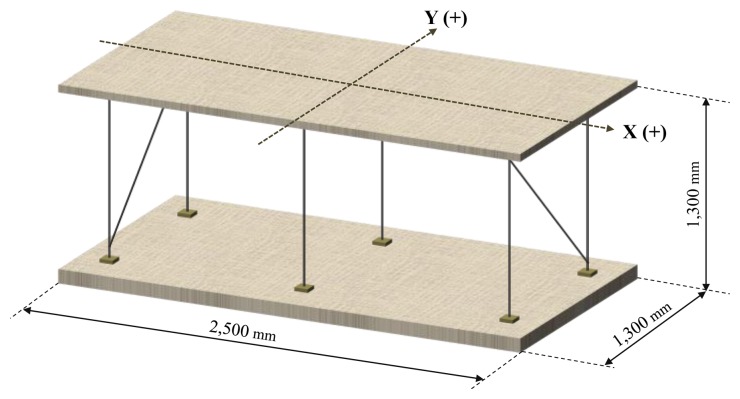
Experimental model.

**Figure 3.**
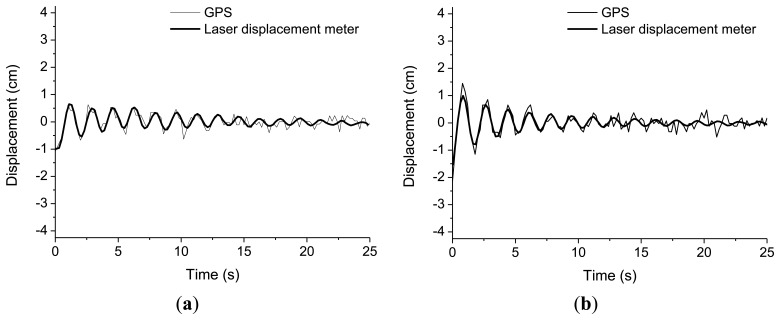

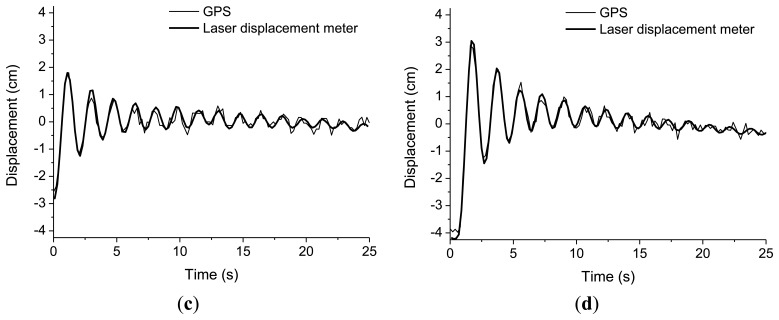
Displacement measurement by GPS and laser displacement meter: (**a**) Initial displacement 1 cm; (**b**) Initial displacement 2 cm; (**c**) Initial displacement 3 cm; (**d**) Initial displacement 4 cm.

The experiment body used was a wooden plate of 2.44 m by 1.24 m while the six vertical elements were D10 rebars (Figure 2). The model was set into free vibration along the X-axis with a given initial displacement. In order to eliminate the Y-axis oscillation component, we installed braces on vertical rebars and shock-absorbent pads on the connection points to reduce energy losses at these points during free vibration. The natural frequency and damping ratio of the model are 0.60 Hz and 1.97%, respectively. The initial displacement of free vibration was varied from 1 cm to 2 cm, 3 cm, then to 4 cm increasingly and measurements were taken at frequency of 5 Hz. Installation of measurement devices and the data acquisition code convention were as shown in Figure 1. The results of this experiment showed, as in Figure 3, that the displacement histories using both the laser meter and GPS coincided, regardless of initial oscillation amplitude. The absolute differences between values measured from GPS and laser displacement meter are summarized in Table 1. It is shown that the maximum difference among the values obtained from the first positive peak to the fifth positive peak is about 4.6 mm.

## 3. Full-Scale Measurement of A 66-Story Building

### 3.1. Building Summary

**Figure 4.**
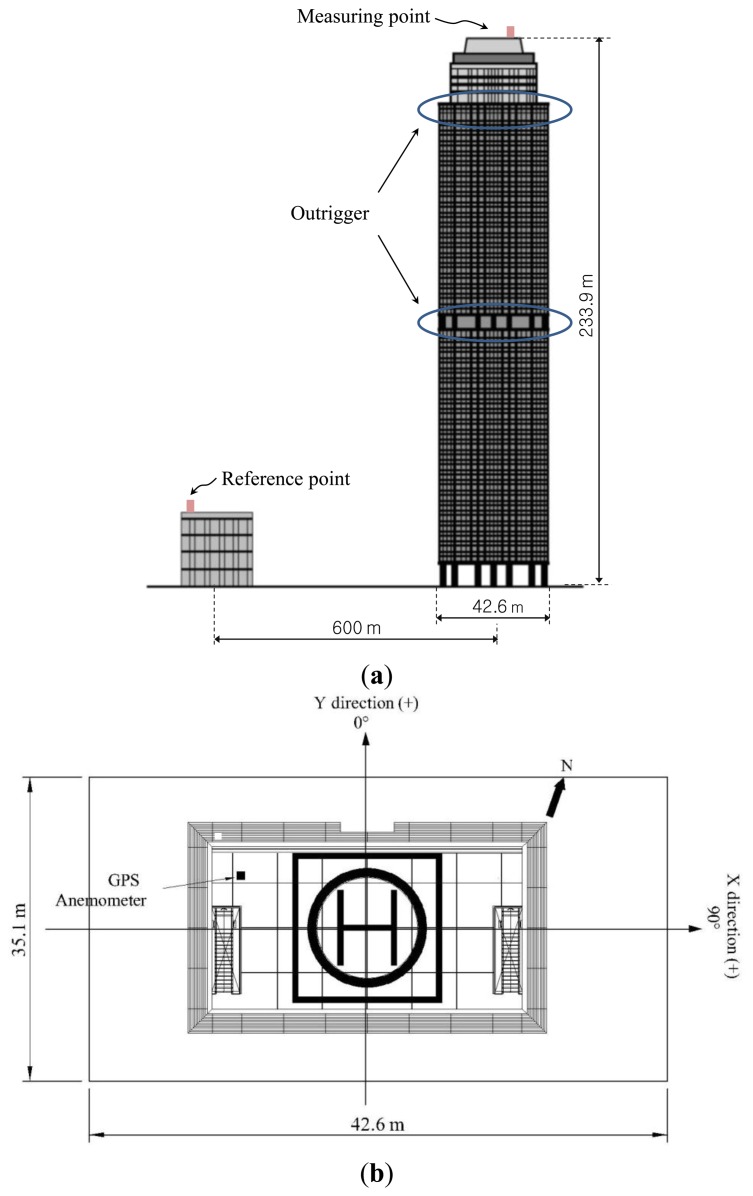
A 66-story building for full-scale test: (**a**) Locations of measuring point and reference point; (**b**) Measurement devices and coordinate convention.

The building used for real measurements is a 66-story multi-purpose facility of reinforced concrete structure with a shear wall and outrigger system (Figure 4). The column-restrained outriggers cause the lateral deflections in the core to be smaller compared to a free-standing core structural system. The delayed joint method is widely used in the field of high-rise buildings with outrigger systems. With this method, the connections of outrigger and columns are fixed after the completion of the construction [30]. Outriggers are located on the 34th and 62th floors. Its height to the heliport where the GPS equipment was installed is 233.9 m and the slenderness ratio is 6.63.

### 3.2. Measurement Equipment

The measurement equipment included GPS antennas for measuring the building's displacement, anemometers, which consist of a wind meter (Model 05103) and a display meter (Model 04503) by Young Inc. (Traverse, MI, USA). The measurable wind speed range was 0 m/s∼60 m/s, wind speed accuracy was ±0.3 m/s and wind direction accuracy ±3.

The GPS equipments used was a Trimble 4700 of 1.22 kg weight, with coarse/acquisition (C/A) code and reflection wave L1 and L2 receiver with automatic on-the-fly (OTF) initialization from five space vehicles (SVs). The antenna was a micro-centered antenna (P/N 14553-01) and the antenna cable was a 10 m low-loss, dedicated antenna cable (P/N 14553-01). The GPS antennas were set on tripods, which were set next to the room parapet.

### 3.3. Measurement Summary

The symbol convention for data acquisition was determined so that the lateral direction was set as the X-axis for data acquisition convenience as shown in Figure 4b. Positions of the measurement devices were as shown in Figure 4b. An anemometer was installed to measure the wind speed and direction at the position on the heliport as shown in Figure 4b. The GPS base station was installed as the reference point on the roof of a five-story apartment building about 600 m away from the measurement building to ensure no lateral displacement (Figure 4a).

Both the base station and rover stations used Trimble's software to take measurements at 5 Hz and the data was stored in the computer. The measurement data was then processed using the Trimble Geometric Office software at 1 Hz. Processed 3-dimensional data displayed in World Geodetic System 1984 (WGS84) coordinates was then transformed into two-dimensional coordinates by establishing a local X-Y coordinate system on the building roof. Data measurements were performed for continuously ten min. To assess the noise level of GPS, the sequential data of 100 measurements under wind conditions with the maximum wind velocity of 0.625 m/s were used. It was shown that the displacement radius on the X-Y plane was approximately 0.34 cm.

### 3.4. Lateral Displacements before and after the Installation of Outrigger Trusses

In order to measure changes in building movements in the construction stage, we monitored the building displacement history before and after installation of the outrigger trusses on the measured building. We did not, however, consider the effect of temperature variations on the displacement. The GPS measurement data was processed at 1 Hz, which is more than twice the natural frequency of the building. Periods of building movement measurements before and after the outrigger truss installation were for seven continuous hours.

**Figure 5.**
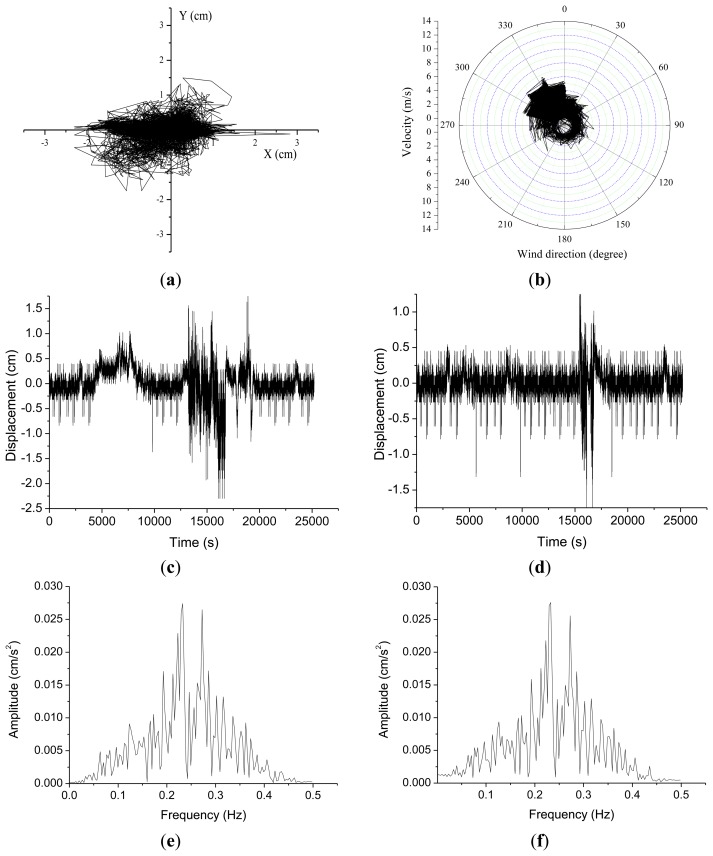
Displacement and wind speed before outrigger truss installation: (**a**) GPS displacement; (**b**) Wind speed and direction; (**c**) Time series in X-direction; (**d**) Time series in Y-direction; (**e**) Fourier spectrum in X-direction; (**f**) Fourier spectrum in Y-direction.

**Figure 6.**
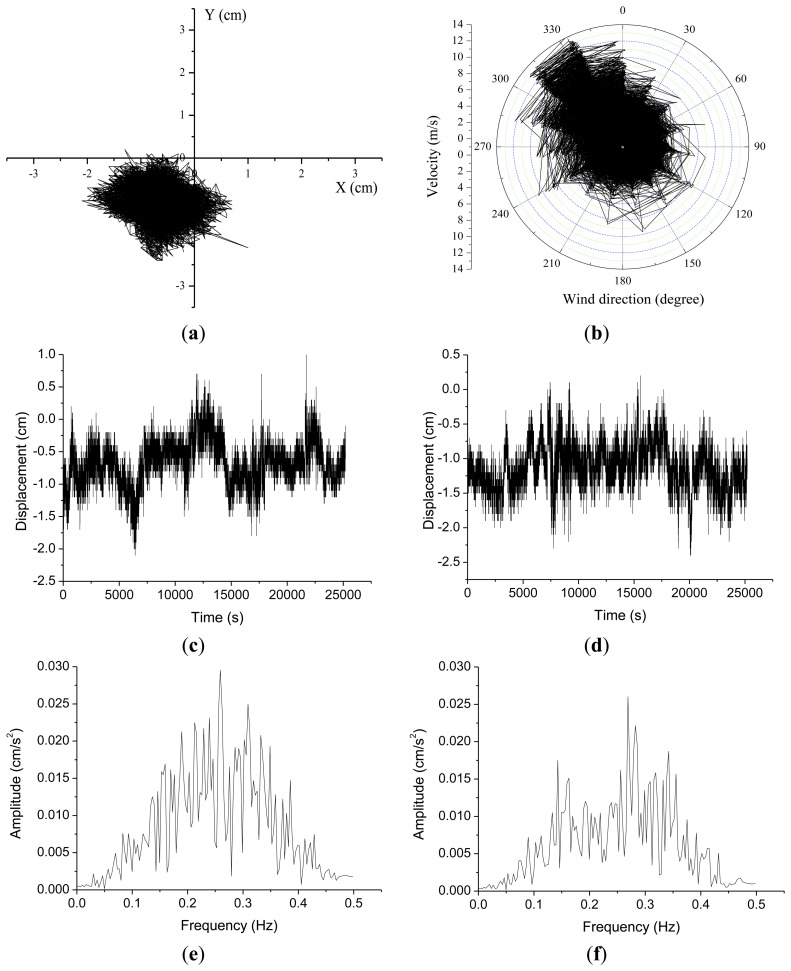
Displacement and wind speed after outrigger truss installation: (**a**) GPS displacement; (**b**) Wind speed and direction; (**c**) Time series in X-direction; (**d**) Time series in Y-direction. (**e**) Fourier spectrum in X-direction; (**f**) Fourier spectrum in Y-direction.

Before the outrigger truss installation GPS measurement of displacements in the X-axis range (Figure 5a) were of −2.3∼+2.83 cm and of −1.75∼+1.5 cm in the Y-axis range. Wind speeds during this time period were as shown in Figure 5b, with a maximum of 6.6 m/s and a mean speed of 2.7 m/s. The natural frequencies of the building in the X and Y axis before the installation of outrigger trusses were 0.232 Hz and 0.233 Hz, respectively. After the outrigger belt truss installation the GPS displacement measurements had an X-axis range of −1.2∼+1.68 cm, and a Y-axis range of −1.58∼+1.32 cm (Figure 6a). The wind speed during this time interval was as shown in Figure 6b, with a maximum of 14.5 m/s and a mean speed of 3.19 m/s. After the installation of outrigger trusses, the natural frequencies of the building in the X and Y axis were found to be 0.263 Hz and 0.271 Hz, respectively. The natural frequencies and Fourier spectrum were obtained from the fast Fourier transform (FFT) analysis of the acceleration data that are computed by numerically differentiating the measured displacements.

As summarized in Table 2, standard deviations were computed to analyze changes in the displacement measurements before and after the outrigger truss installation. Standard deviations before the truss installation were 0.36 cm in the X-direction and 0.22 cm along the Y-axis. After the truss installation the standard deviations were 0.25 cm on the X-axis and 0.26 cm on the Y-axis. One thing to notice is that the relative displacement radius on the X-Y plane was reduced from 0.28 cm before the truss installation to 0.19 cm after the installation. This is an indirect verification of the building's stiffness change from the truss installation in spite of the stronger wind load during measurements after the installation, as shown in Figures 5b and 6b. It is noteworthy that the natural frequencies of the building in X-axis have been changed from 0.23 Hz to 0.26 Hz due to the installation of outrigger trusses. Also, in the Y-axis, the natural frequency has increased from 0.23 Hz to 0.27 Hz.

To evaluate the stiffness changes quantitatively, the ratios of the base shear forces to the roof displacements were used, as shown in Table 3. The base shear forces were calculated based on the assumption that the wind velocity profiles comply with the power law. The 10-min mean wind velocity and relative displacement measured at the top of the building were used. It was shown that the ratios of the base shear forces to the roof displacements in the X- and Y-directions after the outrigger truss installation increased by 122% and 120%, respectively, compared with those before outrigger truss installation.

### 3.5. Displacement Histories for Different Wind Loads

In order to verify the wind speed impact on the building displacement history we selected a 10 min interval from the measurement data when the mean wind speed was maintained steady and divided a 40 s of displacement history into equal intervals of 10 s. Mean wind speed and direction for each interval were as shown in Table 4. The mean wind speed increased from 3.6 m/s, 10.5 m/s, to 13.7 m/s, then decreased to 8.1 m/s. Prevalent wind directions were relatively steady within the range 230°∼265° The mean displacement in Y direction corresponding to static displacement component increased from 3.50 cm, 4.15 cm, 4.30 cm, then decreased to 3.90 cm.

**Figure 7.**
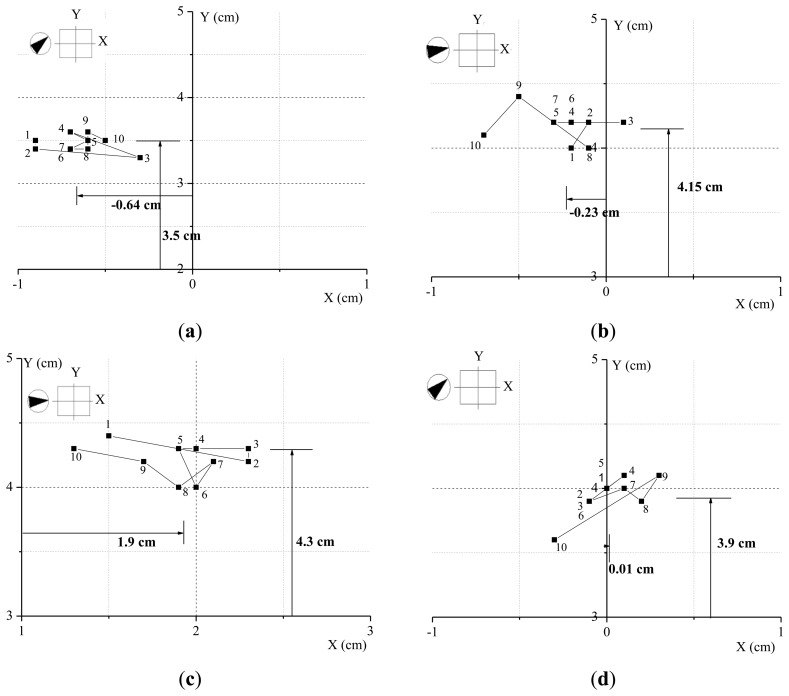
Measured displacements for different mean wind speed at limited time intervals: (**a**) 3.6 m/s; (**b**) 10.5 m/s; (**c**) 13.7 m/s; (**d**) 8.1 m/s.

Displacement history pattern for 10 s, as shown in Figure 7, reveals that as the wind speed increased, directional mean displacements also increased.

The number in Figure 7 is time in seconds. From the figures it may be noted that GPS measurement system is able to accurately resolve movements of a building into static displacement and dynamic fluctuating displacement components.

**Figure 8.**
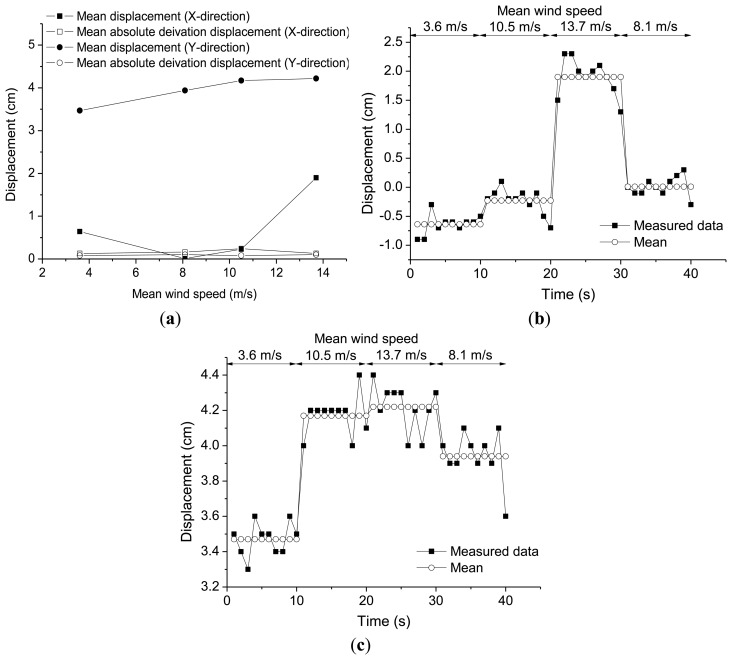
The distributions of the mean displacements and the mean absolute deviation displacements according to the mean wind speeds: (**a**) Distributions of the mean displacements and the mean absolute deviation displacements according to the mean wind speeds; (**b**) Time series in X-direction; (**c**) Time series in Y-direction.

The distributions of the mean displacements and the mean absolute deviation displacements in the X- and Y-directions according to the mean wind speeds are shown in Figure 8. It was shown that the mean displacement components in X- and Y-directions were the predominant components regardless of the mean wind speeds as shown in Figure 8b,c. Standard deviation and range of building displacements as well as the circle rule radius for each time interval were as shown in Table 5.

## 4. Conclusions

In this paper, stiffness changes of a high-rise building due to installation of outrigger trusses, one of the most frequently used lateral load resisting systems for high-rise buildings, have been evaluated by measuring lateral displacements using GPS. To confirm the error ranges of the GPS measurement system used in the full-scale measurement tests, the feasibility of a GPS displacement monitoring system is examined by a free vibration test using an experimental model. It has been confirmed that the two displacement histories of the experimental model obtained from the GPS and a laser measurement device coincided well in the range of displacement greater than 0.5 cm. Finally, the GPS displacement measurement system is applied to monitoring of lateral displacements of a 66-story high-rise building. Based on the translational displacement history monitoring under wind load, it is concluded that indirect verification of the building's stiffness change due to the outrigger truss installation can be done by GPS displacement measurements before and after the installation. The natural frequencies of the building in the X- and Y-directions based on a Fast Fourier Transform (FFT) analysis have been changed from 0.23 Hz to 0.26 Hz and from 0.23 Hz to 0.27 Hz, respectively. It was shown that the stiffness in the X- and Y-directions after the outrigger truss installation increased by 122% and 120%, respectively, compared with those before outrigger truss installation.

## Figures and Tables

**Table 1.** Absolute difference between values (in mm) measured by the GPS and the laser displacement meter (LDM).

**Initial Displacement**		**Positive Peak**					**Average**	**Standard Deviation**

		**1st**	**2nd**	**3rd**	**4th**	**5th**		
1 cm	GPS	6.2	6.3	5.4	5.5	3.5	5.4	1.1
	LDM	6.5	4.9	4.9	5.3	3.2	5.0	1.2
	Absolute difference	0.3	1.3	0.4	0.2	0.4	0.5	0.5

2 cm	GPS	14.5	8.5	6.5	6.6	2.6	7.7	4.3
	LDM	9.9	6.7	4.9	3.7	3.2	5.7	2.7
	Absolute difference	4.6	1.8	1.7	2.8	0.6	2.3	1.5

3 cm	GPS	18.1	8.7	8.4	5.1	3.8	8.8	5.6
	LDM	17.9	11.5	8.5	6.8	5.3	10.0	5.0
	Absolute difference	0.1	2.8	0.1	1.7	1.5	1.3	1.2

4 cm	GPS	28.8	20.0	15.3	8.6	9.9	16.5	8.2
	LDM	30.4	20.3	12.3	10.9	8.6	16.5	8.9
	Absolute difference	1.6	0.4	3.0	2.3	1.3	1.7	1.0

**Table 2.** Displacement changes from outrigger truss installation.

	**Standard Deviation of Displacements (cm)**		**Radius (cm)**

	**X**	**Y**	
Before installation	0.36	0.22	0.28
After installation	0.25	0.26	0.19

**Table 3.** Stiffness changes before and after outrigger truss installation.

	**Before Outrigger**	**Truss Installation**	**After Outrigger**	**Truss Installation**

	**X-Direction**	**Y-Direction**	**X-Direction**	**Y-Direction**
Mean wind velocity	1.23 m/s	2.74 m/s	1.38 m/s	3.43 m/s
Mean roof relative displacement	1.68 mm	1.03 mm	0.95 mm	0.73 mm
Base shear forces	7.59 kN	47.88 kN	9.60 kN	75.10 kN
Stiffness	4.53 kN/mm	46.62 kN/mm	10.06 kN/mm	102.44 kN/mm

**Table 4.** Variation of wind directions for different mean wind speed.

**Wind Speed**	3.6 m/s	10.5 m/s	13.7 m/s	8.1 m/s

**Wind Direction**	230°	260°	265°	260°

**Table 5.** Variation of displacements for different mean wind speed.

**Mean Wind Speed**		**m/s**			

		**3.6**	**10.5**	**13.7**	**8.1**
Standard deviation (cm)	X	0.17	0.21	0.31	0.17
	Y	0.09	0.11	0.12	0.14
Average displacement (cm)	X	−0.64	−0.23	1.90	0.01
	Y	3.50	4.15	4.30	3.90
Displacement range (cm)	X	−0.9∼−0.3	−0.7∼0.1	1.3∼2.3	−0.3∼0.3
	Y	3.3∼3.6	4.0∼4.4	4.0∼4.4	3.6∼4.1
Radius (cm)		0.19	0.24	0.33	0.22
